# Identification and validation of metastasis-related gene ZG16 in the prognosis and progression in colorectal cancer

**DOI:** 10.3389/fonc.2024.1409329

**Published:** 2024-07-24

**Authors:** Yulun Liu, Jie Yang, Wei Han, Tingting Gu, Liqian Yao, Yongqiang Wang, Hua Chen

**Affiliations:** ^1^ Department of General Surgery, Affiliated Kunshan Hospital of Jiangsu University, Kunshan, China; ^2^ School of Medicine, Jiangsu University, Zhenjiang, Jiangsu, China; ^3^ Department of Pathology, Affiliated Kunshan Hospital of Jiangsu University, Kunshan, China

**Keywords:** colorectal cancer, ZG16, metastasis, EMT, Wnt/β-catenin

## Abstract

**Background:**

Metastasis remains the leading cause of mortality among colorectal cancer (CRC) patients. Identification of new metastasis-related genes are critical to improve colorectal cancer prognosis.

**Methods:**

Data on mRNA expression in metastatic and primary CRC was obtained from the Gene Expression Omnibus (GEO) database, including GSE81986, GSE41568, GSE71222, GSE21510, and GSE14333. Additionally, data concerning mRNA expression in colon cancer (COAD) and adjacent normal tissues were acquired from The Cancer Genome Atlas (TCGA) database. Hub genes were identified by weighted gene co-expression network analysis (WGCNA) and differential gene expression analysis. Moreover, we assessed the impact of hub gene expression on both overall survival (OS) and disease-free survival (DFS) in patients and identified ZG16 as a potential target. We generated CRC cell lines transfected with lentivirus OE-ZG16 to investigate proliferation, invasion, and migration *in vitro*. To further elucidate the involvement of ZG16, we utilized gene set enrichment analysis (GSEA) to identify enriched pathways, which were subsequently validated via Western blot analysis.

**Results:**

Five datasets containing primary and metastatic CRC samples from GEO database and CRC samples from TCGA database were included in this study and 29 hub genes were identified by WGCNA and differentially expressed gene (DEG) analysis. Low expression of the hub genes (CLCA1 and ZG16) was associated with poor DFS and OS. We confirmed the low expression of ZG16 in CRC using external database and IHC analysis at both transcriptional and protein levels. In addition, the expression of ZG16 was notably elevated in NCM460 cells in comparison to CRC cell lines. The overexpression of ZG16 in CRC cells has been shown to inhibit the proliferation, invasion, and migration of CRC cells. Furthermore, the overexpression of ZG16 has been found to suppress the activation of the epithelial-mesenchymal transition (EMT) and Wnt/β-catenin signaling pathways in CRC.

**Conclusion:**

ZG16 may serve as a promising therapeutic target for metastatic CRC treatment.

## Introduction

Colorectal cancer is a major malignant tumor endangering global health, and metastasis is the main cause of mortality (1). While radical surgery significantly improves outcomes in the early stages of CRC, the lack of effective treatments remains a key factor contributing to the bleak prognosis of metastatic CRC (mCRC), resulting in a discouraging 5-year survival rate of less than 10% (2). Notably prone to distant metastasis, colorectal cancer frequently disseminates to the liver, a pivotal determinant in the prognosis of primary cancer patients. Individuals facing liver metastases from CRC confront a six-month life expectancy in the absence of intervention (3). Hence, it is crucial to understand the complex molecular mechanisms behind CRC metastasis. Such insights are crucial for advancing the development of more sophisticated and efficacious therapeutic strategies.

The susceptibility and incidence of CRC are due to a variety of causes, including genetic abnormalities, environmental factors, and so on. In recent years, numerous high-throughput techniques, such as microarray gene expression profiling and RNA sequencing (RNAseq), have been employed in the quest for genes underlying common diseases (4). Through bioinformatics analysis, numerous complex molecular and genomic alterations in CRCs were identified. However, a bottleneck arises in their application, given that these techniques typically provide a multitude of candidate genes associated with the investigated disease. Addressing this challenge, network-based approaches have emerged to efficiently discern disease-associated genes within established biomedical networks. Weighted Gene Co-expression Network Analysis (WGCNA) represents a method for clustering genes based on expression patterns (5). WGCNA has found widespread application in bioinformatics research, particularly in the investigation of prognostic markers and therapeutic targets across various tumors and diseases (6).

Here, we used transcription data from Gene Expression Omnibus (GEO) databases and The Cancer Genome Atlas (TCGA) databases for differentially expressed genes (DEGs) analysis and WGCNA hub module gene analysis. The hub genes that were associated with metastasis in CRC were then identified. Notably, it was revealed that the expression of ZG16 exhibited a negative correlation with OS and DFS among CRC patients.

Lectins are a particular group of proteins that can bind to glycans without involving the immune system (7). To be more specific, lectins play a crucial role in the primary defense against infections, cellular transport, immune system modulation, and the prevention of autoimmune diseases (8). Human zymogen granule protein 16 (ZG16), a protein found in human zymogen granules, contains a lectin domain similar to Jacalin and is primarily synthesized by cells that secrete mucus, specifically intestinal goblet cells (9). The expression of the ZG16 gene and the presence of Copy Number Variations (CNVs) have been shown to be associated with a diverse range of molecular and clinicopathological features in CRC, including microsatellite instability (MSI) and MLH1 gene silencing (10). Lower levels of ZG16 in colorectal cancer may indicate a poor prognosis for survival (11). It has been demonstrated that miR-196a regulates ZG16 expression and that ZG16 deletion causes dryness and development of CRC, implying that ZG16 works as a tumor inhibitor (12). The loss of ZG16 may encourage bacterial infiltration of the host system and create local inflammation, which in turn increases the risk of cancer formation (13). Furthermore, ZG16p overexpression dramatically decreased Caco-2 cell proliferation (14). Nonetheless, the precise molecular mechanism of ZG16 in colorectal cancer remains obscure.

The differential expression of ZG16 was confirmed through analysis of external databases and internal clinical samples. Subsequent experimental investigations were conducted to scrutinize the impact of ZG16 on the progression of CRC, with a particular emphasis on assessing its influence on proliferation, invasion, and migration, as well as its role in the epithelial-mesenchymal transition (EMT) process and the Wnt/β-catenin signaling pathway. These analyses provided valuable evidence to elucidate the complex mechanism by which ZG16 is associated with CRC metastasis, and to uncover potential biomarkers for CRC prognosis and identify new therapeutic targets.

## Methods

### Data filtering and processing

In this study, five datasets containing primary and metastatic CRC samples were extracted from the GEO (https://www.ncbi.nlm.nih.gov/gds) database using the R package GEOquery. The datasets included GSE81986 (183 primary and 390 metastasis samples), GSE41568 (80 primary and 39 metastasis samples), GSE71222 (26 primary and 126 metastasis samples), GSE21510 (47 primary and 76 metastasis samples), and GSE14333 (61 primary and 229 metastasis samples), totaling 1257 samples for further analysis.

In addition, CRC samples containing RNA-seq data were obtained from TCGA (https://portal.gdc.cancer.gov/) database using the TCGAbiolinks R package.

### Weighted gene co-expression network analysis

To enhance the precision of network construction, the WGCNA genes were filtered. To conduct WGCNA across the gene expression data profiles of TCGA and GEO database, the WGCNA package in R was utilized. This resulted in the highly co-expressed genes being grouped into modules. PickSoft Threshold is used to create a scale-free network. After calculating the Pearson correlation for all pairs of genes, a matrix of similarities was created. The adjacency matrix was subjected to a transformation process resulting in the creation of a topological overlap matrix (TOM) and dissimilarity matrix, which were subsequently utilized for hierarchical clustering. Subsequently, to determine the constituent modules of the co-expression network, we compared the module attributes calculated earlier with clinical traits.

### Differentially expressed gene analysis

The R package limma was applied to analyze the differentially expressed genes (DEGs) in normal and tumor tissues from the TCGA database, as well as patients with various statuses (primary or metastatic) from the GEO database. The DEGs were subjected to screening based on the criteria |logFC| ≥ 1.0 and adj. P-value < 0.05. T Visualization of the results were achieved through the generation of volcano plots and Venn diagrams using the ggplot2 and Venn Diagram R packages.

### Gene expression analysis and survival analysis

GEPIA2.0 (http://gepia2.cancer-pku.cn/) was used to evaluate the impact of hub gene expression on patients’ overall survival (OS) and disease-free survival (DFS). The TIMER2.0 database (http://timer.cistrome.org/) was utilized to assess the variances in ZG16 expression between pan-cancerous tissues and surrounding normal tissues. The TNM plot database (https://www.tnmplot.com/) and the Human Protein Atlas (https://www.proteinatlas.org) were used to validate ZG16 expression at mRNA and protein levels.

### Gene set enrichment analysis

According to the median value of gene expression, 650 CRC samples from TCGA were divided into the ZG16-high expression group and the ZG16-low expression group. DEGs between the ZG16-high expression group and the ZG16-low expression group were identified using the R package DESeq2. The enrichment of the relevant pathways with ZG16 was identified using GSEA and assessed for statistical significance with adjusted p-values <0.05 and false discovery rate <0.25.GSEA analysis was performed using the R package clusterProfiler.

### Tissue microarray and immunohistochemistry

Tissue microarrays were created by extracting dual 1mm diameter cores from 156 colorectal cancer patient samples, including 136 pairs of tumor tissues and adjacent normal tissues. Immunohistochemistry (IHC) research was conducted using a human anti-ZG16 antibody (1:200; Solarbio, China) on formalin-fixed, paraffin-embedded tissue microarrays. The level of ZG16 staining was assessed using a specified computational method: The percentage of positively stained tumor cells (0, ≤ 5% positive cells; 1, 5–25% positive cells; 2, 26–50% positive cells; 3, 51–75% positive cells; 4, ≥ 75% positive cells) is multiplied by the staining intensity score (0, no staining; 1, weak staining, light yellow; 2, moderate staining, yellow-brown; 3, strong staining, brown) to obtain a final score between 0 and 12. A final score of 4 or below is classified as indicative of low expression, while a score between 6 and 12 is considered indicative of high expression. All patients provided informed consent, and the study was approved by the Medical Ethics Committee of the Affiliated Kunshan Hospital of Jiangsu University, adhering to the principles outlined in the Declaration of Helsinki. The Ethic Approval number is MR-32-23-036448.

### Cell lines and culture

NCM460, a normal cell line of the human colon epithelium, along with the colon cancer cell lines HCT116, HT29 and SW480, were acquired from Kunshan Hospital’s Central Laboratory. Cells cultured in DMEM medium with 10% fetal bovine serum and 1% penicillin-streptomycin. ZG16-overexpressed HCT116 and SW480 virus-packaging cells were cultured in the same medium with 4 g/mL puromycin. The cells were grown in an environment with 5% CO_2_ that was humidified at 37 degrees Celsius. All experiments were repeated independently three times.

### Cell transfection

GeneChem (Shanghai, China) supplied the OE-ZG16 human lentivirus. Lentivirus was used to infect HCT116 cells, with an MOI of 10 serving as the standard. Lentivirus was used to infect SW480 cells with an MOI of 20 serving as the standard. All of the cells were infected with the use of transfection reagents called HiTransG P (GeneChem), and then they were treated with a medium that included puromycin for a period of three weeks. This allowed for the generation of cells that consistently overexpressed ZG16.

### Transwell assay

*In vitro*, the migratory and invasive properties of cells were evaluated using Transwell experiments. 1×10^5^ CRC cells were cultured in a serum-free medium within a Transwell plate’s upper chamber (24-well, 12 mm, Corning, Life Sciences, USA), while the lower chamber contained 20% FBS for the migration assay. Following a period of 36-48 hours of incubation, the cells that had migrated were immobilized, treated with a solution of crystal violet at a concentration of 0.05%, and counted in five randomly selected areas utilizing fluorescence microscopy. For the invasion test, a chamber was prepared with 20μg of Matrigel (BD Biosciences, San Diego, California, United States), and 1×10^5^ CRC cells were placed in it. After 36-48 hours, the invaded cells were fixated, stained with a crystal violet solution containing 0.05%, and then viewed under an inverted microscope.

### Wound healing assay

A density of 1 x 10^5^ cells per well was chosen for the planting of the cells in the 6-well plates. Once the cellular density reached 70%, the cells were removed in a straight line using a blue 1-ml micropipette tip. After flushing the detached cells with PBS, a serum-free medium was added to the cell culture medium. Fluorescence microscopy was used to compare the widths at 0, 12, 24 and 48h to determine the distance of migration.

### Cell viability assay

The tumor cells were grown in 96-well plates with smooth bottoms. At varying times after transfection (0, 12, 24, 48, and 72 h), 10μL of CCK-8 solution (Beyotime, China) was injected into each well. After incubation for 4 hours, an optical value (OD) at 450 nm of absorbance was measured using a microplate reader. This experiment was conducted with three independent replicates.

### Colony formation assay

HCT116 cells and SW480 cells, which had been treated with trypsin, were placed in a 6-well plate with a density of 500 cells per well. Following a two-week incubation in culture, the CRC cells were subsequently stained using crystal violet and fixed using methanol. After allowing the samples to dry in the air, we manually tallied the number of colonies that contained over 50 cells. The experiments were conducted independently in three repetitions.

### EdU assay

The 5-Ethynyl-2’-deoxyuridine (EdU) assay was carried out with the assistance of an EdU assay kit (RiboBio, Guangzhou, China) in accordance with the instructions provided by the manufacturer. At a density of 4 × 10^3^ cells per well, the cells were grown in 96well plates. Following a culture that lasted for twenty hours, the cells underwent treatment with 50 mol/L Edu and incubated at 37°C for a total of two hours. The cells were fixated for 20 minutes with 4% paraformaldehyde, additional 20 minutes of permeabilization with 0.5% Triton X‐100, then incubated for 30 minutes at room temperature with 100 μLof1×Apollo^®^ reaction cocktail. At the end of the experiment, 100 L of Hoechst 33342 (5 g/mL) stained cell nuclei for 20 minutes, and the results were observed using fluorescence microscopy.

### RNA isolation and RT-qPCR

Total RNA was extracted using the RNeasy mini kit (Qiagen, Valencia, CA), following the manufacturer’s instructions. All RNA samples’ concentration and purity were determined using an absorbance ratio of 260/280 nm. Following that, 1μg of RNA was reverse-transcribed using Bio-Rad’s iScriptTM cDNA Synthesis kit (Hercules, CA). The UltraSYBR Mixture reagent (CWBIO, Jiangsu, China) and the iCycler thermal cycler were utilized for real-time PCR analysis. Normalization with GAPDH was used to determine the relative mRNA expression level of a gene. The primers that were utilized for real-time quantitative real-time PCR were designed by RIBOBIO company.

### Western blotting

Using a cell lysis solution, the cells were extracted and lysed. BCA reagent was used to determine the concentration of extracted total proteins. Using SDS-PAGE electrophoresis, proteins were analyzed. They were electrotransferred onto a PVDF membrane and then washed with TBS for 15 minutes. It was blocked, and then the necessary primary antibodies were added. The GAPDH gene served as a control and they were subjected to overnight incubation at a temperature of 4°C. The second antibody was incubated on the membrane for 2 hours at room temperature. A more advanced chemiluminescent reagent was utilized for band identification. An enhanced chemiluminescence reagent was used to identify the bands. ImageJ (NIH, Bethesda, Maryland, United States) was used to determine the grey values of the protein bands. The final values shown are the averages obtained from three different measurements taken independently.

### Statistical analysis

In our data, the IBM SPSS 22.0 software was used to analyze the statistical differences. At least three times each *in vitro* experiment was conducted independently. We used the Student’s t-test or ANOVA to analyze group differences. All information is presented as the mean standard deviation. A p-value of less than 0.05 was regarded as statistically significant.

## Results

### Identification and prognostic value of the hub genes by integrated bioinformatics analysis

**Figure 1:**
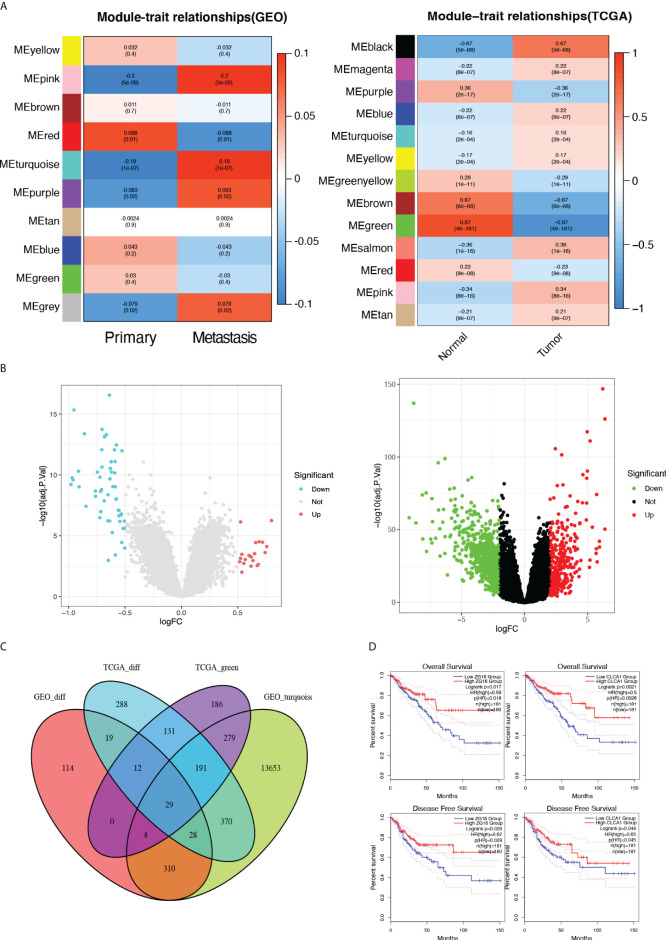
Identification of DEGs by Integrated Bioinformatics Analysis. **(A)** The correlation heatmap of gene modules with the clinical factors of colon cancer. **(B)** Volcano plot of DEGs in the five GEO and the TCGA dataset. **(C)** The Venn diagram of genes among DEG lists and co-expression modules. In total, 29 overlapping genes in the intersection of DEG lists and two co-expression modules. **(D)** Based on the GEPIA2 database, the Hub gene ZG16 and CLCA1 were positively correlated with both overall survival and disease-free survival in CRC (P < 0.05).

To identify metastasis related gene sets for CRC, we performed a WGCNA analysis from the GEO datasets (GSE81986, GSE41568, GSE71222, GSE21510, and GSE14333) as well as the TCGA database based on the WGCNA package (**Supplementary Figure 1**). Here, 10 modules in the above five GEO datasets and 13 modules in TCGA datasets were identified (**Figure 1A**), and heat maps of module-trait relationships indicated significant distinctions in the turquoise modules within the GEO dataset when comparing primary and metastatic individuals (r=0.18, p=1e-07). Furthermore, the heat maps also demonstrated a pronounced difference in the green modules from the TCGA dataset, when distinguishing between normal and tumor individuals (r=0.87, p=8e-161). A total of 15,696 co-expressed genes were extracted from highlighted modules, including 14,864 genes from the turquoise module and 832 genes from the green module. Utilizing the limma package with a cut-off criteria of adj.p<0.05 and |logFC|≥1.0, a total of 516 differentially expressed genes (DEGs) were identified from five GEO datasets and 1,068 DEGs from TCGA. (**Figure 1B**). By taking the intersection of the co-expression genes and the DEGs, 29 Hub genes were finally obtained (**Figure 1C**). Based on the GEPIA2 database, we found the Hub gene ZG16 and CLCA1 were positively correlated with both overall survival and disease-free survival in CRC (P < 0.05) (**Figure 1D**, **Supplementary Figure 2**).

### Validation of ZG16 expression and its association with clinicopathological features in CRC

**Figure 2:**
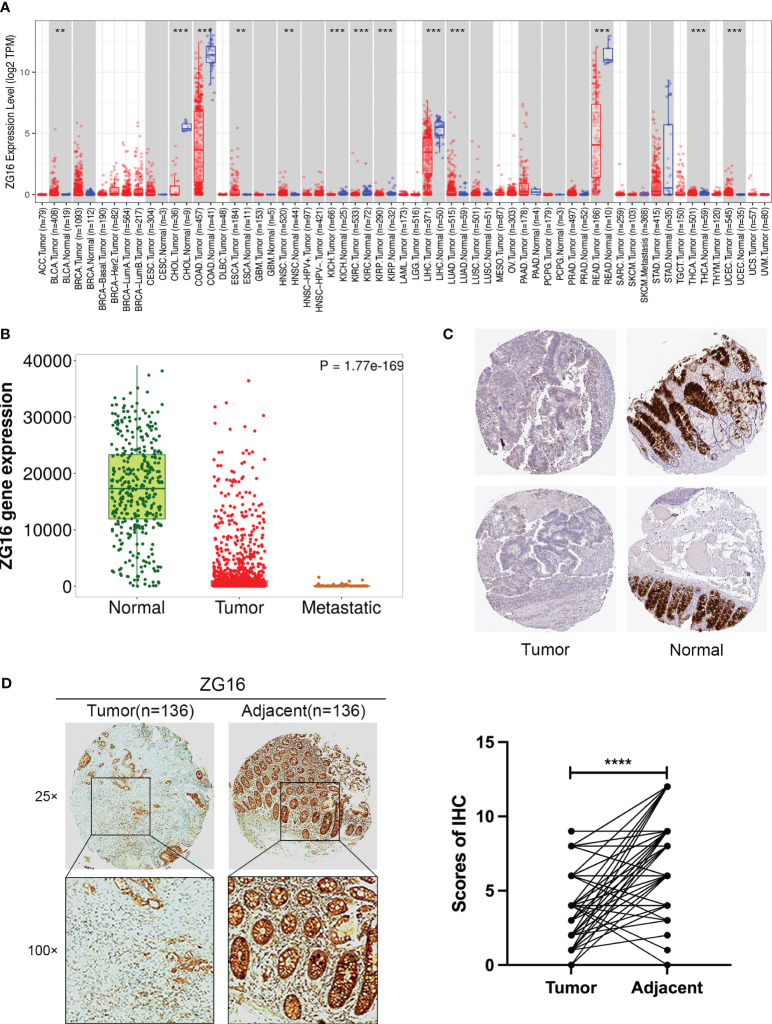
Validation of ZG16 expression and its association with clinicopathological features in CRC. **(A)** ZG16 expression in different cancers from TIMER2.0. **p < 0.01; ***p < 0.001. **(B)** The mRNA expression levels of ZG16 were significantly lower in metastatic and tumor tissues. **(C)** The protein expression levels of ZG16 were significantly decreased in HPA database. **(D)** IHC experiment was carried out to show that the ZG16 protein expression was downregulated in 136 CRC tumor tissues compared with the paired adjacent-normal tissues. ****p < 0.0001.

It has been reported that CLCA1 can suppress CRC aggressiveness (15), whereas the the role and mechanisms of ZG16 in CRC metastasis remain poorly understood. We further confirmed that ZG16 expression was downregulated in tumor tissues compared to normal tissues, especially in colon cancer (COAD) and rectal cancer (READ) in TIMER database (**Figure 2A**). Then, the TNM plot database showed that the mRNA expression levels of ZG16 were significantly lower in metastatic and tumor tissues and the protein expression levels of ZG16 were significantly decreased in HPA database (**Figures 2B, C**). We also detected the expression of ZG16 in several CRC cell lines and NCM460 cell lines (**Supplementary Figure 3**). Furthermore, IHC experiment was carried out to show that the ZG16 protein expression was downregulated in 136 CRC tumor tissues compared with the paired adjacent-normal tissues (**Figure 2D**). According to the median score of ZG16 staining intensity, 156 CRC patients were classified into ZG16-high (70 cases) and ZG16-low (86 cases) and the expression of ZG16 was revealed to be associated with T, N and AJCC TNM stage (**Table 1**).

**Table 1:** Correlation between ZG16 expression and clinicopathological characteristics of CRC patients.

Characteristics	High	Low	P value
**Total No.**	70	86	
Gender, n (%)			0.641
Male	36 (51.4%)	41 (47.7%)	
Female	34 (48.6%)	45 (52.3%)	
Age, n (%)			0.985
< 60	17 (24.3%)	21 (24.4%)	
≥ 60	53 (75.7%)	65 (75.6%)	
T.stage, n (%)			0.020*
T1-2	16 (22.9%)	8 (9.3%)	
T3-4	54 (77.1%)	78 (90.7%)	
N.stage, n (%)			0.047*
N0	38 (54.3%)	33 (38.4%)	
N1/2	32 (45.7%)	53 (61.6%)	
M.stage, n (%)			0.618
M0	68 (97.1%)	81 (94.2%)	
M1	2 (2.9%)	5 (5.8%)	
AJCC.stage, n (%)			0.023*
Stage I/II	38 (54.3%)	31 (36%)	
Stage III/IV	32 (45.7%)	55 (64%)	

*p < 0.05

### ZG16 inhibits the proliferation of CRC cells *in vitro*

**Figure 3:**
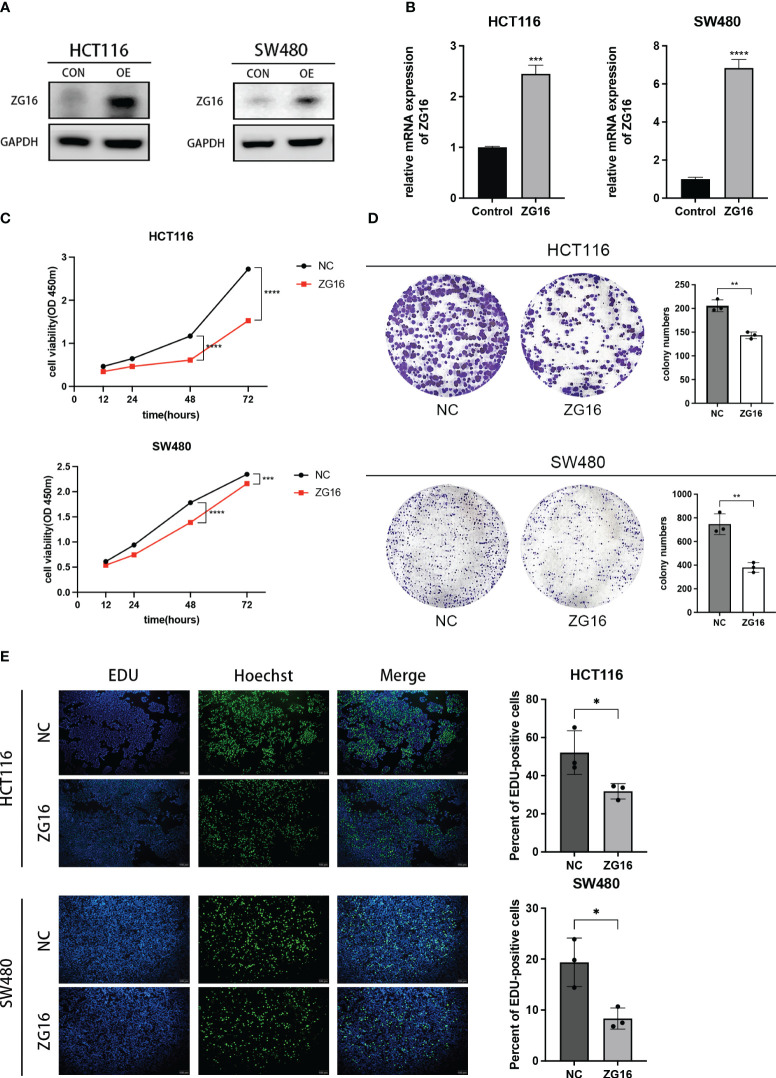
ZG16 inhibits the proliferation of CRC cells *in vitro*. **(A)** Western blotting analysis of HCT116 and SW480 cells with ZG16-overexpression. **(B)** RT-QPCR analysis of HCT116 and SW480 cells with ZG16-overexpression. **(C)** CCK8 assay showed the effect of ZG16 on the proliferation rate of HCT116 and SW480 cells. Data were expressed as mean ± SD of three independent experiments. **(D)** Colony formation assay showed the effect of ZG16 on the colony-forming ability of HCT116 and SW480 cells. **(E)** The EdU incorporation assay showed the effect of ZG16 on the colony-forming ability of HCT116 and SW480 cells. *p < 0.05; **p < 0.01; ***p < 0.001; ****p < 0.0001.

Here, we choose HCT116 and SW480 cells to construct the ZG16 stably over-expressed cell lines. The transfection efficiency was verified by western blot and qPCR (**Figures 3A, B**). The CCK-8 assay was employed to assess the impact of ZG16 on the proliferative capacity of CRC cells. The results revealed that growth rate of HCT116 and SW480 cells over-expressed ZG16 was significantly decreased compared with the control group (**Figure 3C**). Consistently, colony formation assay suggested that over-expression of ZG6 inhibited the colony formation rate (**Figure 3D**). Moreover, the EdU incorporation assay showed the ZG16 overexpression group’s EdU-positive cells were significantly fewer than those in the control group (**Figure 3E**). Taken together, these data indicated that ZG16 suppressed cell proliferation in CRC cells.

### ZG16 inhibits the migration and invasion of CRC cells via regulating EMT

**Figure 4:**
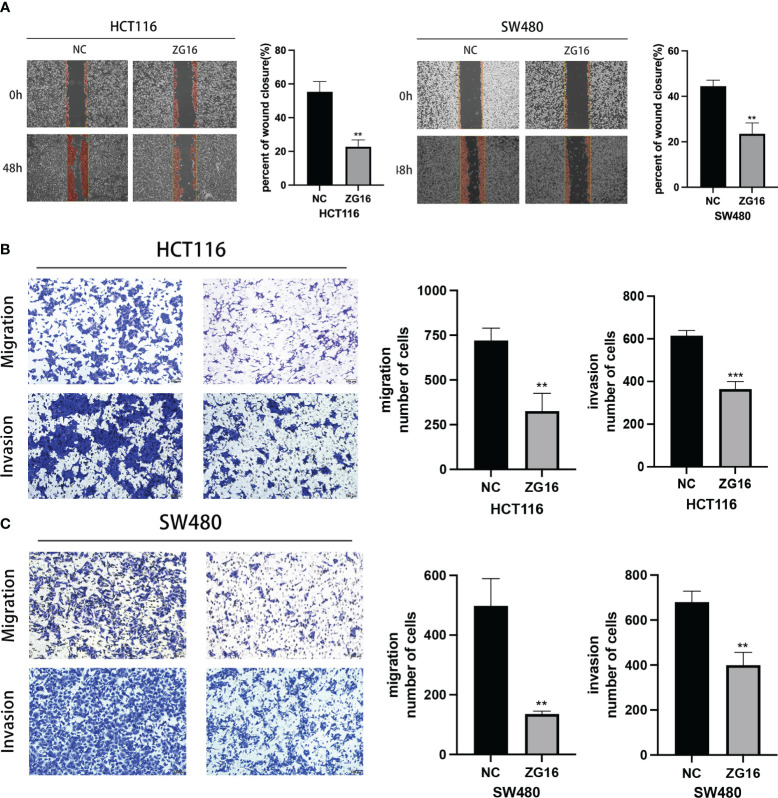
ZG16 inhibits the migration and invasion of CRC cells via regulating EMT. **(A)** Cell migration ability of HCT116 and SW480 cells with ZG16-control or ZG16-overexpression was proved by wound healing assay. **(B, C).** The effects of ZG16 overexpression on CRC cell migration and invasion were assessed by transwell assay. The data were represented as mean ± SD. **p < 0.01; ***p < 0.001..

**Figure 5:**
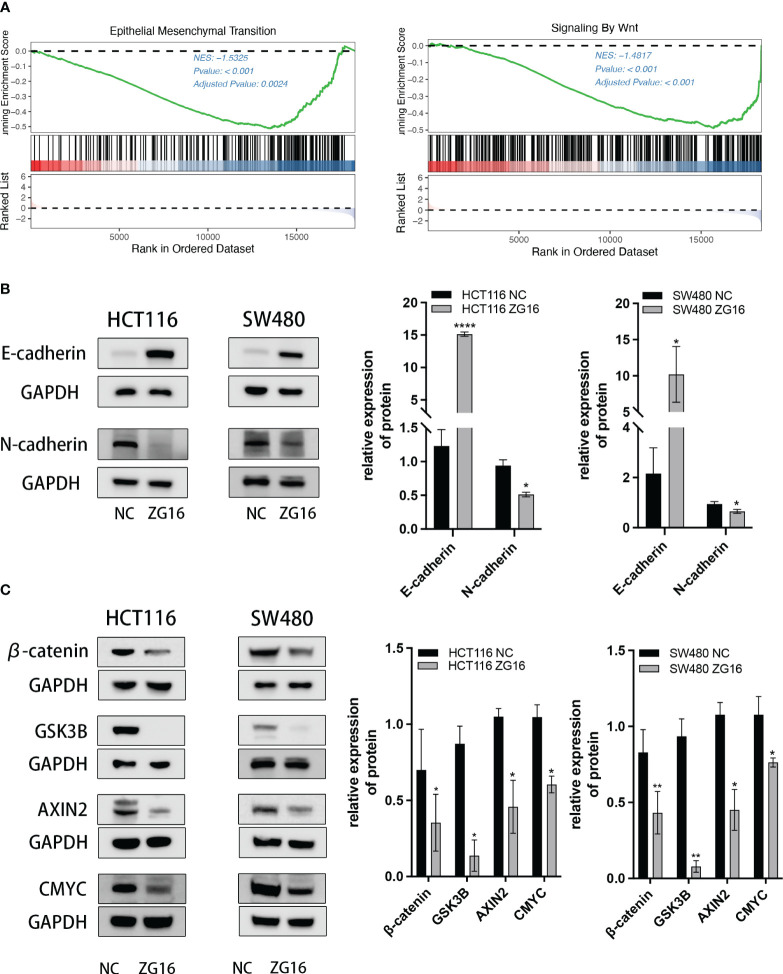
ZG16 regulates the EMT via Wnt/β-catenin signaling pathway. **(A)** GSEA analysis showed EMT and Wnt signaling enrichment in the ZG16-low-expression group of CRC patients. **(B)** Western blot analysis of E-cadherin and N-cadherin levels in CRC cells with or without ZG16-overexpression. **(C)** Western blot analysis of β-catenin, Gsk3β, Axin2, and c-myc levels in CRC cells with or without ZG16-overexpression. The data were represented as mean ± SD. *p < 0.05. **p < 0.01; ****p < 0.0001.

As shown above, ZG16 was identified as a Metastasis-Related gene in CRC, so we further performed experiments to validate its association with CRC cell migration and invasion. The results demonstrated that up-regulation of ZG16 inhibited the migration of HCT116 and SW480 cells through wound healing assays (**Figure 4A**). The HCT116 and SW480 cells exhibited reduced migratory and invasive capabilities in the transwell assays, both with and without Matrigel when ZG16 was over-expressed (**Figures 4B, C**). In addition, GSEA analysis on TCGA datasets indicated ZG16 was involved in the EMT process (**Figure 5A**). Due to the crucial role of EMT in CRC metastasis, we detected the effects of ZG16 on EMT represent markers by western blot, which showed that overexpression of ZG16 in HCT116 and SW480 cells led to elevated levels of E-cadherin, a marker of epithelial cells, and reduced levels of N-cadherin, a marker of mesenchymal cells (**Figure 5B**). These results indicated ZG16 could regulate the EMT to inhibit the migration and invasion of CRC cells.

### ZG16 regulates the EMT via Wnt/β-catenin signaling pathway

The above results indicated that ZG16 could regulate the EMT in CRC cells, and GSEA analysis also showed a significant Wnt signaling enrichment in the ZG16-low-expression group of CRC patients (**Figure 5A**). Given the important role of Wnt/β-catenin signaling pathway in EMT (16, 17), the expression of downstream protein in HCT116 and SW480 cells with stable ZG16 overexpression were determined by western blot. As we expected, overexpression of ZG16 remarkably attenuated the expression of β-catenin, Gsk3β, Axin2, and c-myc (**Figure 5C**). Taken together, these results indicated that ZG16 could regulate EMT through Wnt/β-catenin signaling pathway.

## Discussion

According to statistical surveys, the prognosis of metastatic colorectal cancer (mCRC) is poor, with less than a 20% 5-year survival rate, due to the dearth of an effective treatment (2). In addition, malignant colon tissue does not respond to chemotherapy drugs in advanced stages (18). Significantly, colorectal cancer is characterized by a notable level of tumor diversity and a substantial amount of genetic instability, both of which greatly influence the patient’s treatment and prognosis (19). As a result, finding new biomarkers to help us comprehend the molecular alterations that cause colorectal cancer is essential for developing efficient early-detection methods and new targeted treatments. New technologies can aid in the identification of essential genes associated with the development of colorectal cancer. The primary genes are regarded as potential biomarkers in the progression of colorectal cancer. Hence, the examination of the underlying genes associated with colorectal cancer may precede the evaluation of biomarkers. High-throughput sequencing technology’s quick progress allows us to quickly extract a lot of genetic information from samples of patients, which can help us comprehend the fundamental mechanisms and identify new molecular targets for the therapy of CRC.

Through analysis of the co-expression genes and differentially expressed genes (DEGs) in the GEO and TCGA datasets, a total of 29 metastasis-related hub genes were identified. Subsequent OS and DFS analyses indicated that decreased expression levels of CLCA1 and ZG16 were significantly associated with a worse prognosis in CRC.

ZG16 (Zymogen Granule Protein 16) was discovered in 1998 as a linker molecule in pancreatic acinar cells and is expressed in digestive system mucus-secreting cells such as the liver, colon, small intestine, and pancreas (9). It has been suggested that the dominant role in regulating intestinal immune activity is played by the goblet cells in the intestine, which primarily express ZG16, as demonstrated (20). In a study comparing ZG16^-/-^ and ZG16^+/+^ mice, it was discovered that ZG16 can collect Gram-positive bacteria and inhibit germs from penetrating mucus (21). As a result, ZG16 may be a crucial element of the mucus barrier, and its presence enables bacteria to stay at a safe distance from the surface of epithelial cells. Consequently, ZG16 might serve as an essential component of the mucus barrier, allowing bacteria to maintain a secure distance from the epithelial cell surface. Meanwhile, the existence of a jacalin-like lectin domain in ZG16 indicates that the human counterpart might have a significant role in the immunotherapy of colon cancer (13). ZG16 contains a lectin-like region that can activate T cells in the digestive system’s immune system and contribute to tumor recognition. Natural killer cells, which are a subset of lymphocytes responsible for enhancing anti-tumor immunotherapy and regulating inflammation and autoimmune disorders, belong to the innate immune system of the body (22). Previous study suggests that ZG16 inhibits PD-L1 expression and enhances NK cell survival and growth, potentially regulating the intestinal immune system through goblet cells (10).

Previous research has indicated that the secreted protein ZG16 is down-regulated in liver cancer (23), and CRC patients who have distant metastasis have a further reduction in ZG16 expression (12). ZG16 loss is also an early event in the development of CRC, as seen in the transition from adenomatous polyps to adenocarcinoma (24). In this study, the relative expression of ZG16 in three CRC cell lines (HCT116, SW480, and HT29) and a normal colonic epithelial cell line (NCM460), revealing downregulation of ZG16 in the CRC cell lines. Subsequent analysis of the effects of ZG16 on colorectal cancer cell lines demonstrated that overexpression of ZG16 reduced the proliferation, migration, and invasion of CRC cells.

Epithelial tumors go through a crucial process called epithelial-mesenchymal transition (EMT). EMT is essential for vigorous cell movement during embryonic development, however, tumor cells can reactivate the EMT program, thereby increasing tumor cell invasiveness and promoting tumor recurrence, metastasis, as well as treatment resistance (25). The classical epithelial markers E-cadherin and N-cadherin are key components of intercellular adhesion junctions and are the most prominent targets of EMT (26, 27). In this study, we constructed a ZG16 lentivirus stable cell line to detect EMT-related proteins, and the results showed that overexpression of ZG16 increased E-cadherin expression and decreased N-cadherin expression. Therefore, we hypothesized that ZG16 may inhibit the EMT process in CRC cells, thereby inhibiting CRC invasion and metastasis. The invasion and metastasis of colorectal cancer are related to many factors. Studies have shown that the dysregulation of Wnt/βcatenin, p53, TGF-β/smad, NF-κB, Notch, VEGF, and JAKs/STAT3 pathways in colorectal cancer leads to the occurrence and progression of colorectal cancer (28). Mutations or dysregulation of the β-catenin destruction complex, consisting of APC, Axin2, CK1, and GSK-3β, lead to the activation of Wnt signaling (29–31). Additionally, an increased nuclear β-catenin level is recognized as a characteristic feature of invasive colorectal cancer (CRC), resulting in the activation of Wnt-associated targets such as c-myc, thereby enhancing cell proliferative, invasive, and migratory capabilities (32, 33). GSK3 is a kinase originally isolated from rat skeletal muscle that phosphorylates and inactivates glycogen synthase (34). Studies have shown that the expression of GSK3β is closely related to the occurrence and prognosis of colorectal cancer (35). Silencing or pharmacological inhibition of GSK3β in osteosarcoma cells leads to apoptosis (36). Our research findings indicate that the upregulation of ZG16 led to decreased expression levels of c-myc, GSK-3β, and Axin2 compared to the control group, as determined by Western blot analysis. These results offer additional support for the role of ZG16 as a negative regulator of the Wnt signaling pathway.

In conclusion, our study demonstrates that ZG16 inhibits proliferation, migration, and invasion by suppressing EMT and Wnt signaling activity.

## Data availability statement

The datasets presented in this study can be found in online repositories. The names of the repository/repositories and accession number(s) can be found in the article/**Supplementary Material**.

## Ethics statement

The studies involving humans were approved by Medical Ethics Committee of Kunshan Hospital Affiliated to Jiangsu University. The studies were conducted in accordance with the local legislation and institutional requirements. The human samples used in this study were acquired from gifted from another research group. Written informed consent for participation was not required from the participants or the participants’ legal guardians/next of kin in accordance with the national legislation and institutional requirements.

## Author contributions

YL: Data curation, Validation, Writing – original draft, Formal Analysis, Investigation, Visualization. JY: Data curation, Validation, Writing – original draft, Formal Analysis, Investigation, Visualization. WH: Data curation, Writing – review & editing. TG: Data curation, Writing – review & editing. LY: Data curation, Writing – review & editing. YW: Conceptualization, Funding acquisition, Writing – review & editing, Resources. HC: Conceptualization, Funding acquisition, Writing – review & editing, Resources.

## Supplementary material

Supplementary Figure 1 Analysis on GEO (GSE81986, GSE41568, GSE71222, GSE21510, GSE14333) and TCGA data using WGCNA package.

Supplementary Figure 2 Overall survival and disease-free survival of the Hub genes in CRC based on GEPIA2 database.

Supplementary Figure 3 The expression of ZG16 in several CRC cell lines and NCM460 cell lines.
