# Supplementary figures and images for: Identification and validation of metastasis-related gene ZG16 in the prognosis and progression in colorectal cancer
Source: Front Oncol. 2024 Jul 24;14:1409329. doi: 10.3389/fonc.2024.1409329 (PMC11303331; doi:10.3389/fonc.2024.1409329)

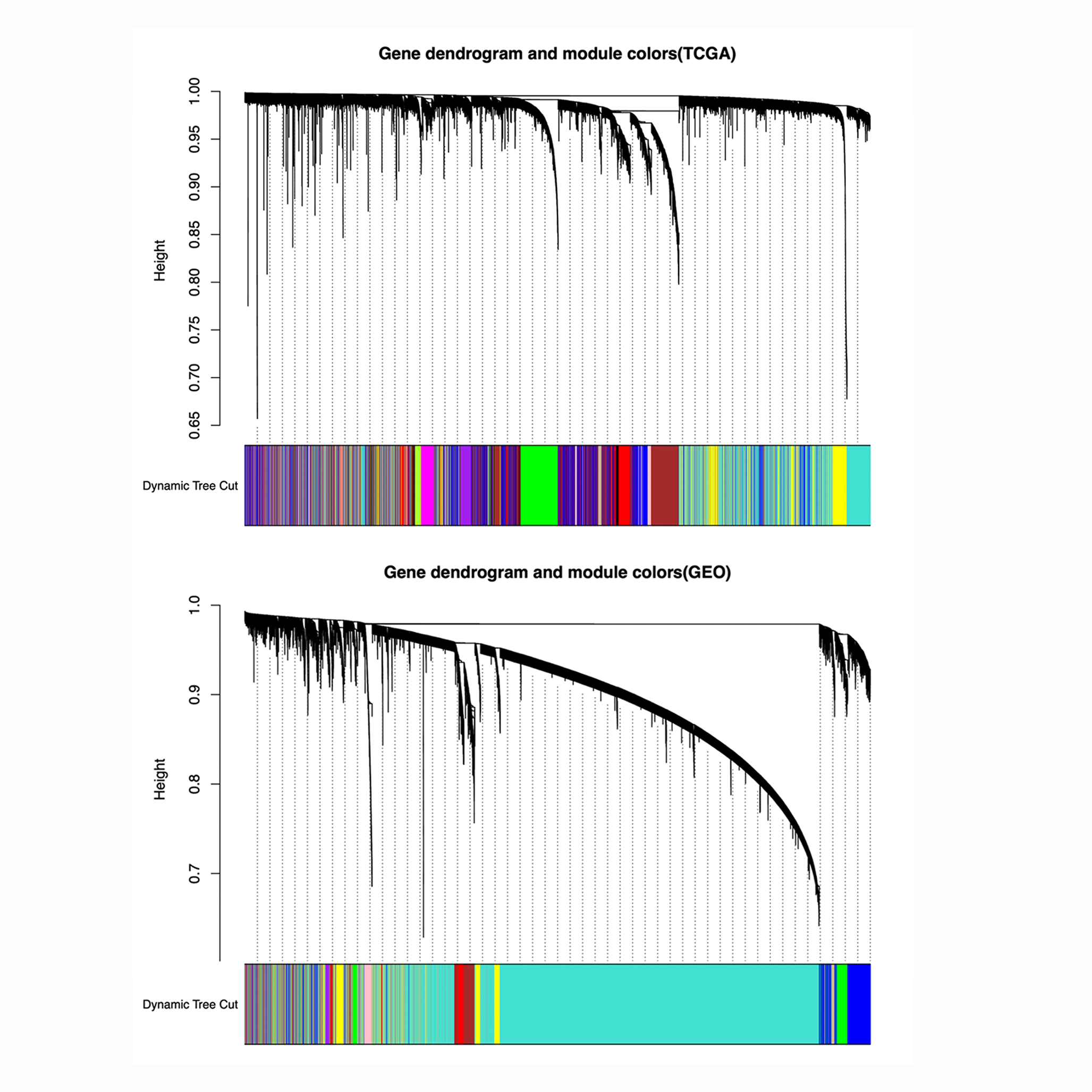

Supplement: Supplementary Figure 1 — Analysis on GEO (GSE81986, GSE41568, GSE71222, GSE21510, GSE14333) and TCGA data using WGCNA package. [file Image_1.tif]

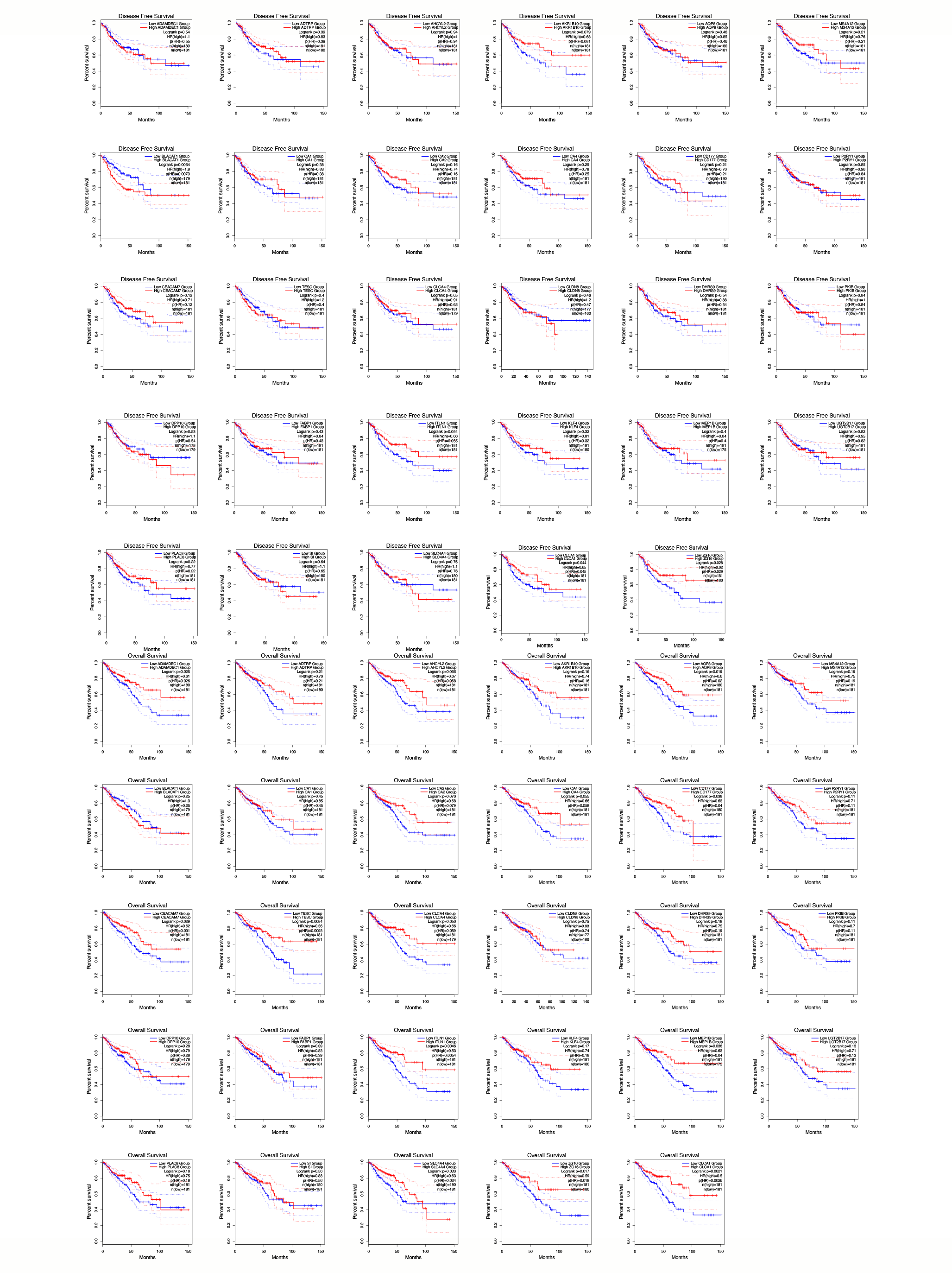

Supplement: Supplementary Figure 2 — Overall survival and disease-free survival of the Hub genes in CRC based on GEPIA2 database. [file Image_2.tif]

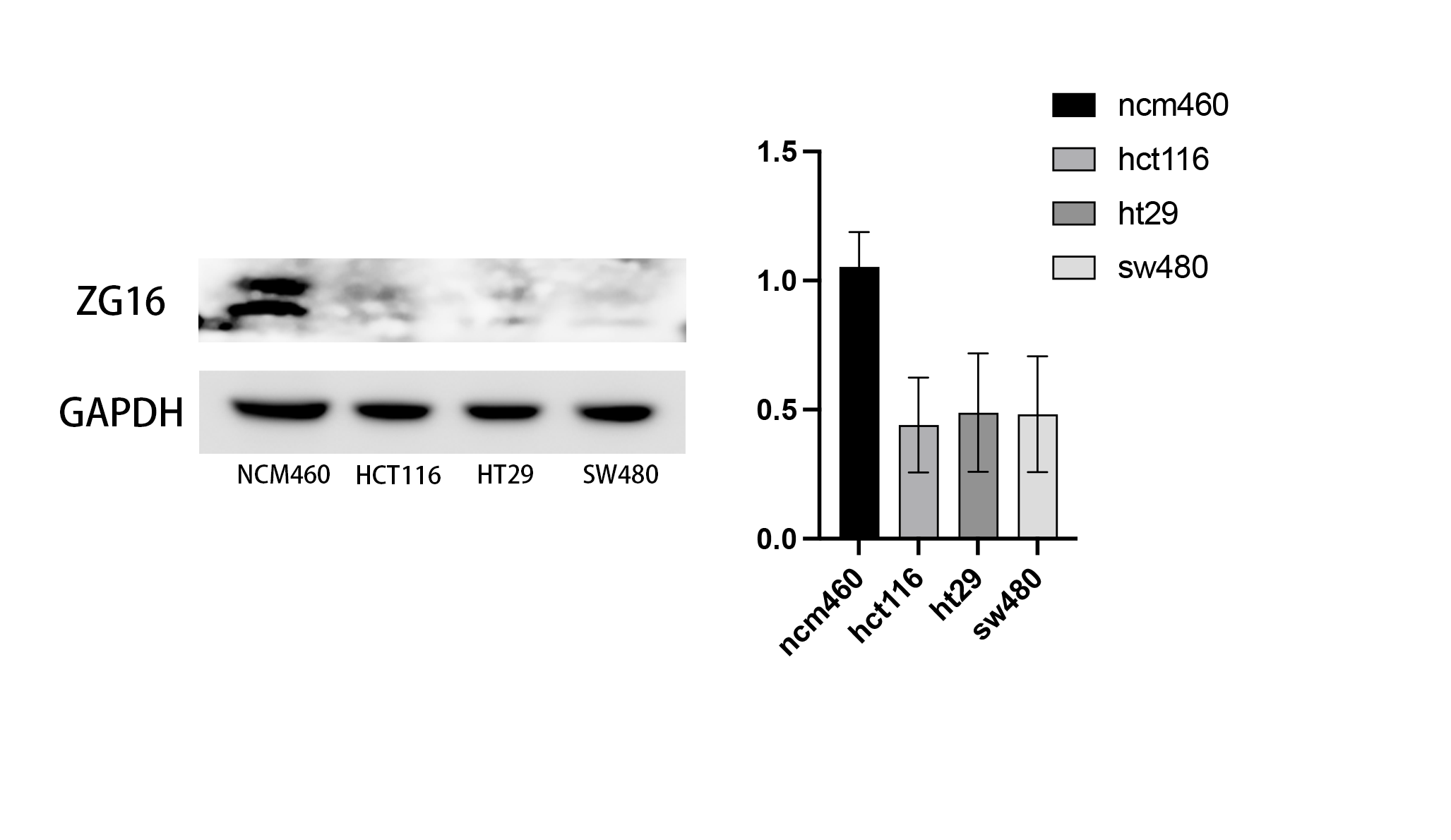

Supplement: Supplementary Figure 3 — The expression of ZG16 in several CRC cell lines and NCM460 cell lines. [file Image_3.tif]
